# Vacuole and Mitochondria Patch (vCLAMP) Protein Vam6 Is Involved in Maintenance of Mitochondrial and Vacuolar Functions under Oxidative Stress in *Candida albicans*

**DOI:** 10.3390/antiox10010136

**Published:** 2021-01-19

**Authors:** Xiaolong Mao, Li Yang, Yingzheng Liu, Congcong Ma, Tianyu Ma, Qilin Yu, Mingchun Li

**Affiliations:** Key Laboratory of Molecular Microbiology and Technology, Ministry of Education, College of Life Science, Nankai University, Tianjin 300071, China; fudan671@163.com (X.M.); 18783958821@163.com (L.Y.); liuwenbo129@163.com (Y.L.); mcmacongcong@163.com (C.M.); matianyu3317658@163.com (T.M.); yuqilin7007@163.com (Q.Y.)

**Keywords:** *Candida albicans*, vCLAMPs, oxidative stress, Vam6

## Abstract

*Candida albicans* is one of the most common opportunistic fungal pathogens in human beings. When infecting host cells, *C. albicans* is often exposed to oxidative stress from the host immune defense system. Maintenance of mitochondrial and vacuolar functions is crucial for its resistance to oxidative stress. However, the role of vacuole and mitochondria patchs (vCLAMPs) in cellular oxidative stress resistance and in the maintenance of organelle functions remains to be elucidated. Herein, the function of the vCLAMP protein Vam6 in response to oxidative stress was explored. The results showed that the *vam6*∆/∆ mutant exhibited obvious mitochondrial swelling, mtDNA damage, reduced activity of antioxidant enzymes, and abnormal vacuolar morphology under H_2_O_2_ treatment, indicating its important role in maintaining the structures and functions of both mitochondria and vacuoles under oxidative stress. Further studies showed that deletion of *VAM6* attenuated hyphal development under oxidative stress. Moreover, loss of Vam6 obviously affected host tissue invasion and virulence of *C. albicans*. Taken together, this paper reveals the critical role of vCLAMPs in response to oxidative stress in *C. albicans*.

## 1. Introduction

*C. albicans* is a common opportunistic fungal pathogen in human beings [1]. Generally, *C. albicans* exists in a benign symbiotic state and does not show pathogenicity in healthy humans [2]. However, the mortality rate is reported to be 40% for patients with candidemia or invasive candidiasis [3]. *C. albicans* infections are thought to be caused by efficient escape from oxidative attacks of the immune system [4]. During invasion of host cells, the primary stress that *C. albicans* encounters is oxidative agents generated by host immune cells [4,5]. At the beginning of invasion, *C. albicans* first responds to external oxidative stress by rapid induction of gene expression involved in ROS scavenging [6]. However, when *C. albicans* successfully infects host tissues, its oxidative response system is instead in an inhibited state at the transcriptional level. After *C. albicans* is swallowed into macrophages, on the one hand, it can resist oxidative stress through morphological transformation [7], while on the other hand, it secretes extracellular antioxidant enzymes to resist oxidative stress. *C. albicans* can secrete a variety of antioxidant enzymes to the cell surface, the most important of which is superoxide dismutase (SOD). Several studies have suggested that vacuoles are quite important in promoting mycelial development and improving antioxidative stress [8,9,10]. Additionally, many studies have attempted to elucidate the nature of mitochondrial production of reactive oxygen species (ROS) and the target of subsequent oxidative damage [11]. Generally, there is a balance between ROS generation and elimination. Once imbalanced, it may lead to the accumulation of ROS in mitochondria, and virulence will decrease [12]. The above studies showed that the maintenance of mitochondrial morphology and function is essential for the survival of eukaryotic cells under oxidative stress [13]. However, the role of the vacuole and mitochondria patchs (vCLAMPs) in the oxidative response is not adequately understood.

In Saccharomyces cerevisiae, Vps39 is a membrane protein that belongs to the VPS (Vacuolar Protein Sorting) series. ScVps39 forms a complex with Ypt7, which is necessary for vacuolar protein transport and vesicle transport. In addition, ScVps39 plays an important role in the early vacuole fusion process and maintains the vacuole morphology. Recent research found that ScVps39 combines with Ypt7 and Tom40 to form mitochondrion and vacuole patches (vCLAMPs) [14,15,16]. Through vCLAMPs [15], the liposomes and ions between mitochondria and vacuole are exchanged [14,15]. However, the ScVps39 homologue Vam6 has never been studied in *C. albicans*.

In this study, we found that *C. albicans* possesses the homolog protein of the S. cerevisiae vCLAMP protein ScVps39 [17], named Vam6. A *VAM6* deletion mutant was constructed by PCR-mediated two-step homologous recombination. Further investigations showed that Vam6 is required for maintaining vacuole morphology and function. Moreover, the *vam6*∆/∆ mutant exhibited obvious mitochondrial dysfunction, increased ROS levels, and reduced SOD1 activity under oxidative stress conditions. These results indicated that Vam6 was necessary in maintaining vacuolar and mitochondrial functions under the oxidative stress response. Further research revealed that deletion of *VAM6* led to abnormal hyphal development under oxidative stress, and to attenuated host invasion and virulence. Taken together, these results revealed the role of vCLAMPs in oxidative stress response, thereby providing new avenues for treatment of *C. albicans*-associated infections.

## 2. Materials and Methods

### 2.1. Strains and Plasmids Construction

The strains used in this article are listed in Table S1. All *C. albicans* strains were generated in the BWP17 background. The primers are listed in Table S2. The *vam6::ARG4* cassette was amplified by T100 Thermal Cycler (BIO-RAD Laboratories, Hercules, CA, USA) using the primer pair of the VAM6-5DR and VAM6-3DR from the plasmid pRS-ArgΔ*Spe*I. Secondly, we used pDDB57 plasmid as a template to amplify *URA3* fragment, and obtained the *vam6::URA3* gene box. The homozygous mutant (*vam6::ARG4 /VAM6*) was identified by PCR. The optimal amplification program was one cycle of 5 min at 94 °C; 30 cycles of 30s at 94 °C, 45 s at 52 °C, and 3 min at 72 °C; one cycle of 10 min at 72 °C. A wild-type band of 3.5 kb and an *ARG4* band of 2.4 kb are shown in the Figure S1A. The homozygous mutant strains (*vam6::ARG4/vam6:: URA3*) were identified by PCR. Only the *ARG4* band of 2.4kb and *URA3* band of 2.1kb were found (Figure S1B). Finally, *vam6*Δ/Δ was selected on SC medium (0.67% yeast nitrogen base without amino acids, 2% glucose, 0.2% complete amino acid mixture, adding 80 μg/mL uridine) [18]. To obtain *vam6*Δ/Δ+*VAM6* (Figure S1E), the pDDB78-*VAM6* plasmid was transformed into *vam6*Δ/Δ after *Nru* I digested. At the same time, the location of plasmid pAu34M-*VAM6-GFP* was linearized by *Bgl* II restriction endonuclease, and transformed into WT to obtain WT-*VAM6-GFP* strain. 

To assay the virulence of *C. albicans*, a 4.9 kb *Bgl* II-*Pst* I fragment from pLUBP (encoding *URA3* and its adjacent gene, *IRO1*) was introduced into the WT, *vam6*Δ/Δ, and *vam6*Δ/Δ+*VAM6* mutant, obtaining the strains WT^a^, *vam6*Δ/Δ^a^ and *vam6*Δ/Δ+*VAM6*^a^ with *URA3* reintroduced to their common locus.

### 2.2. Fluorescence Microscopy

Fluorescence microscopy was used to observe the location of Vam6-GFP in *C. albicans*. WT-*VAM6-GFP* cells were cultured in a liquid medium, adjusted to an OD_600_ of 0.1 in SC medium, cultured for 4 h, and then harvested. After MitoTracker Red (dissolved in dimethyl sulfoxide (DMSO), final concentration: 1 mmol/mL) staining, the cells were observed by fluorescence microscope [19]. The vacuoles were observed after FM4-64 (N-(3-triethylammoniumpropyl)-4-(6-(4-(diethylamino) phenyl) hexatrienyl) pyridinium dibromide) (dissolved in dimethyl sulfoxide (DMSO), final concentration: 50 μg/mL; Sigma, St. Louis, MO, USA) staining [20] in the same way.

### 2.3. Flow Cytometry

To measure the mitochondrial membrane potential (MMP), cells were cultured in YPD medium (containing 5 mm/L H_2_O_2_) for 1 h. Then cells were incubated with a cationic lipid fluorescent dye (JC-1, dissolved in DMSO, final concentration: 1 μg/mL; Sigma-Aldrich, St. Louis, MO, USA) at 30 °C for 30 min, and the cells were measured by flow cytometry (FACS Calibar flow cytometry, BD, San Jose, CA, USA).

### 2.4. Electron Microscopic Observation

Transmission electron microscopy was used to view the vacuole and mitochondria patch (vCLAMP). The strains were cultured in YPD (containing 5 mM H_2_O_2_) for 1 h, and then harvested, washed twice by PBS. The cells were suspended in 2.5% glutaraldehyde fixative and fixed at 4 °C for 12 h. Then cells were washed three times by PBS. The cells were then collected by centrifugation, 1% osmic acid was added, and the cells were suspended and fixed at room temperature for 1 h. The cells were collected by centrifugation and washed three times by PBS for 10 min each time. Cells were fixed by gradient ethanol dehydration. The cells were collected by centrifugation and embedded. The embedded samples were cut into 50–100 nm tissue sections using an ultramicrotome. The cuts were then transferred onto a copper mesh and dried. The samples were observed by a transmission electron microscope (Tecnai G2 F-20, FEI Company, Hillsboro, OR, USA).

Immunolabeling was performed using anti-GFP antibody (MBL598, 1:100) for 2 h at room temperature followed by Goat Anti-Rabbit IgG/Gold (10 nm) (Solarbio, 1:100 dilution) for 30 min at room temperature. The samples were further stained with uranium acetate and citric acid, dried, and observed by a transmission electron microscope (FEI Company, Hillsboro, OR, USA).

### 2.5. Determination of Intracellular ATP, Reactive Oxygen Species (ROS) and SOD1 Levels

Intracellular ATP concentration was measured using the ATP-based creatine kinase catalyzing adenosine triphosphate and creatine to produce creatine phosphate, as previously described [21,22]. 

Intracellular ROS concentration was measured using 2′,7′-Dichlorodihydrofluorescein diacetate (DCFH-DA) (Shanghai, China), which is oxidized by intracellular ROS to form fluorescence DCF, as previously described [1,4]. The cells of the experimental group and the treatment group were collected, and the cells were stained with DCFH-DA (final concentration: 10 μmol/mL) and washed by PBS. The fluorescence intensity of the cells was measured by a fluorescent multilabel reader 2300 (Enspire^TM^, Perkin Elmer, Ltd., Singapore).

The activity of SOD1 in the cells was detected using a superoxide dismutase (SOD1) assay kit (Hydroxylamine method) (Nanjing Jiancheng Bio., Nanjing, China). This assay was based on the superoxide anion radical generated by the xanthine and xanthine oxidase reaction system. The latter can oxidize hydroxylamine to form nitrite and appear purple under the action of a color developer [21]. The optical density at 550 nm of the samples was detected by a spectrophotometer (BIO-RAD Laboratories, Hercules, CA, USA).

### 2.6. Hyphal Induction

To explore the role of Vam6 in hyphal development of *C. albicans*, Spider and RPMI-1640 medium (RP MI-1 640 po wd er 1.04%, MO PS 0.418%, pH 7.0) were selected as solid hypha-inducing media. Strains were cultured at 30 °C and 37 °C for six days to observe hyphal development. To further confirm the role of Vam6 in hyphal development, the strains were also cultured in liquid hypha-inducing medium, i.e., the liquid RPMI-1640 medium, and then stained by Calcofluor White (CFW; final concentration: 10 mg/mL) for fluorescent microscopy.

### 2.7. Assay of Macrophage-Killing Capacity

To evaluate the interaction between the murine macrophages and *C. albicans*, the macrophage RAW246.7 cells (obtained from the Cell Resource Center, China Academy of Medical Science, Beijing, China) were suspended in DMEM medium containing 10% fetal bovine serum (FBS), and cultured at 37 °C with 5% CO_2_ concentration for 24 h, so that the cell concentration was about 5 × 10^5^ cells/mL. The log phase of *C. albicans* was suspended in RPMI-1640 medium at a concentration of 1 × 10^6^ cells. After the cell suspension was incubated with macrophages for a certain period, the mortality of *C. albicans* was determined by CFU counting method. The percentage of macrophages damaged by *C. albicans* was determined by the propidium iodide (PI) staining method.

### 2.8. Virulence Assay

All mice used in this study were placed at a constant temperature of 24 ± 2 °C in room, a 12 h light/dark cycle, lighted at 7:00 in the morning, and food and drink at conditions of freedom in Nankai University Medical College. Every effort was made to minimize animal suffering and animal populations.

Ten 4–6-week-old ICR female SPF mice were used per group, obtained from the HKF Bioscience CO., LTD., Beijing, China. The WT^a^, *vam6*Δ/Δ^a^ and *vam6*Δ/Δ+*VAM6*^a^ strains were cultured in YPD medium to 12 h, washed with PBS, and mice were injected through the tail vein (the injection cells were 5 × 10^5^). The death of the mice was recorded after the first day of vaccination. The survival rate of the mice and statistical analysis were performed by the SPSS software (Version 20, IBM, Chicago, IL, USA). On the third day after fungal infection, three mice in each group were sacrificed, and the kidneys were sampled, weighed and homogenized with PBS buffer. The suspensions of kidney homogenates were diluted and plated on the YPD solid media. After two days of incubation at 30 °C, fungal colonies were counted and the results were normalized by kidney weight. At the same time, some kidneys were taken and fixed with 10% formalin for 24 h. After hematoxylin-eosin staining, the kidney sections were observed by a light microscope (BX53, Olympus, Tokyo, Japan) to evaluate the degree of inflammation and tissue damage by the fungal cells. 

## 3. Results

### 3.1. Vam6 Is Critical for Formation of vCLAMPs

**Figure 1:**
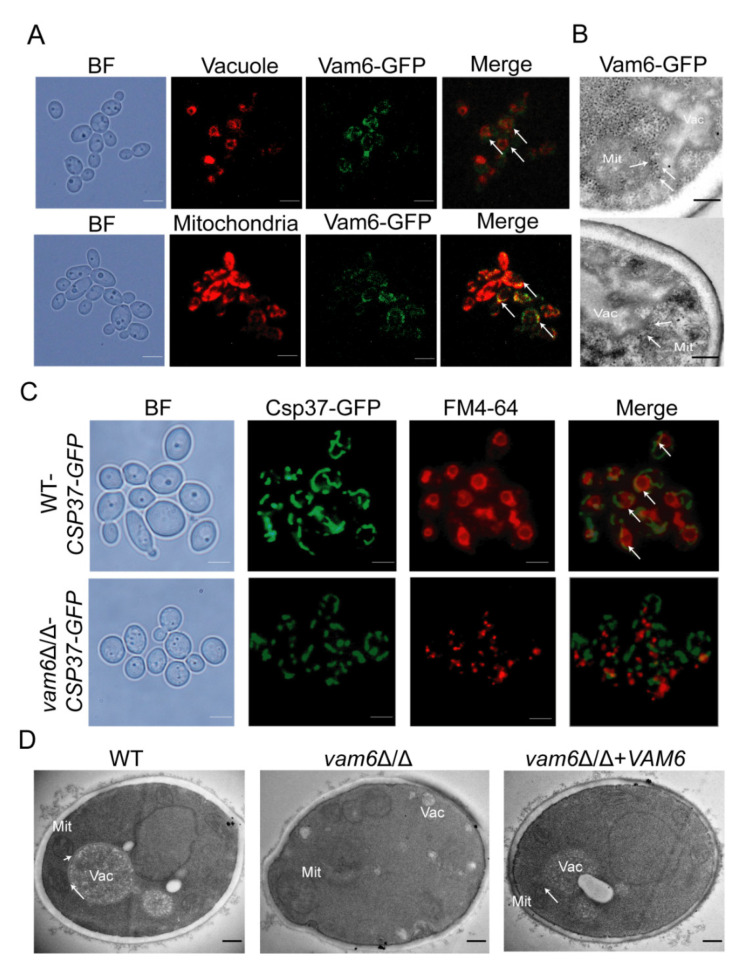
Intracellular localization of Vam6 and its role in formation of vCLAMPs. (**A**) Localization of Vam6 in *C. albicans*. Bar = 5 μm. (**B**) The localization of Vam6-GFP was observed by immuno transmission electron microscope. The Vam6-GFP was visualized using Anti-GFP IgG/Rabbit and Goat Anti-Rabbit IgG/Gold (10 nm). Mit, mitochondria; Vac, vauoles. Bar = 200 nm. (**C**) Colocalization of the mitochondria and the vacuoles in WT and *vam6*Δ/Δ. The white arrows indicate vCLAMPs. Bar = 5 μm. (**D**) TEM images of the cells under oxidative stress. The cells were cultured in liquid YPD containing 5 mM H_2_O_2_ for 1 h, fixed and cut for TEM observation. Bar = 200 nm.

Most proteins of vCLAMPs are located at the mitochondrion-vacuole connecting sites. In this study, Vam6 (orf 19.1567) was found in *C. albicans* through the online NCBI BLASTP software (https://www.ncbi.nlm.nih.gov/Structure/cdd/docs/cdd_search.html), a homologous protein of *S. cerevisiae* Vps39. To determine whether Vam6 is a vCLAMP protein, the cellular location of Vam6 in *C. albicans* was investigated by fluorescent labeling. Fluorescence microscope observation showed that Vam6-GFP was located both on the vacuolar membrane labeled with FM4-64, and on the mitochondria labeled by MitoTracker Red (Figure 1A). Through immunoelectron microscopy (IEM), Vam6 itself accumulated in vCLAMP (Figure 1B). The results indicated that Vam6 may be a protein localized at vCLAMPs.

To study the role of Vam6 in the formation of vCLAMPs, mitochondria were labeled by Csp37-GFP, a fusion protein localized on the outer membrane of mitochondria in *C. albicans* (Figure S2), could be used as a mitochondria marker [22], and vacuoles were labeled by FM4-64 in wild-type (WT) and *vam6*Δ/Δ strains. Fluorescence microscopy showed that the WT strain had abundant mitochondrion-vacuole colocalizing sites (indicated by white arrows, Figure 1C, up), indicating normal formation of vCLAMPs in the WT cells. In contrast, *vam6*Δ/Δ showed no obvious mitochondrion-vacuole colocalizing sites (Figure 1C, bottom), suggesting a severe defect in formation of vCLAMPs in the mutant. Transmission electron microscopy (TEM) observation further confirmed that the WT and reconstituted strains had regular mitochondria adhering to the vacuoles, while the mutant showed fragmented mitochondria not contacting the vacuoles (Figure 1D). These results revealed that Vam6, as a vCLAMP-localizing protein, is required for normal formation of vCLAMPs in *C. albicans*.

### 3.2. Deletion of VAM6 Leads to Hypersensitivity to Oxidative Stress

**Figure 2:**
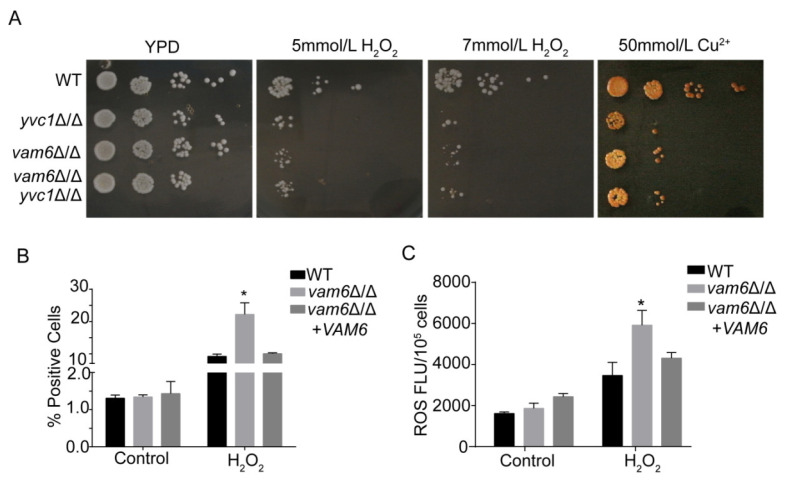
Hypersensitivity of the *vam6*Δ/Δ mutant to oxidative stress. (**A**) Cells were overnight cultured in liquid YPD, spotted on YPD plates containing 5 mM H_2_O_2_, 7 mM H_2_O_2_, or 50 mmol Cu^2+^. The plates were then cultured and photographed. (**B**) Percent of dead cells under treatment of 5 mM H_2_O_2_ for 1 h. The treated cells were stained by PI and observed by fluorescence microscopy for calculation of PI-positive cells. (**C**) Intracellular ROS levels of the strains revealed by DCFH-DA staining. * indicates significant difference between the mutant and the control strains (*p* < 0.05).

Yvc1, one of the calcium signaling members, plays a critical role in cell tolerance to oxidative stress. The *yvc1*∆/∆ strain is always used as an oxidative stress sensitive strain [1]. In the process of infecting the host, oxidative stress is an inevitable challenge for *C. albicans*. To further investigate the role of Vam6 in *C. albicans* cellular processes, sensitivity of the *vam6*Δ/Δ mutant to oxidative stress was evaluated in H_2_O_2_-containing plates. When cultured in YPD contained 5 mM or 7mM H_2_O_2_, growth of *vam6*Δ/Δ was significantly inhibited as compared to the WT strain (Figure 2A). Similar growth inhibition was observed in the *yvc1*∆/∆ strain, an oxidative stress-sensitive strain [1]. Furthermore, in order to detect whether cell death was caused by exogenous oxidative stress, the dead cells were stained by propidium iodide (PI). Consistently, the mutant had much higher death rate than the WT and reconstituted strains under oxidative stress (>20% versus <10%) (Figure 2B). Moreover, ROS assays with 2′, 7′-dichlorodihydrofluorescein diacetate (DCFH-DA) staining revealed that the mutant exhibited higher intracellular ROS levels than the control strains (Figure 2C).

Ion homeostasis is closely involved in intracellular redox homeostasis, and disturbance in ion homeostasis may result in oxidative stress [4]. Plate growth assays further showed that the *vam6*Δ/Δ mutant, similar to *yvc1*Δ/Δ, exhibited hypersensitivity to 50 mmol/L of Cu^2+^ (Figure 2A). However, as compared to WT and *yvc1*Δ/Δ, *vam6*Δ/Δ was not sensitive to Ca^2^^+^, Mn^2+^, Mg^2+^ and Zn^2+^ (Figure S3). Taken together, loss of Vam6 rendered the cells hypersensitive to oxidative stress, indicating a critical role of Vam6 in oxidative stress resistance in this pathogen.

### 3.3. Deletion of VAM6 Affects Vacuolar Morphology and Function under Oxidative Stress

**Figure 3:**
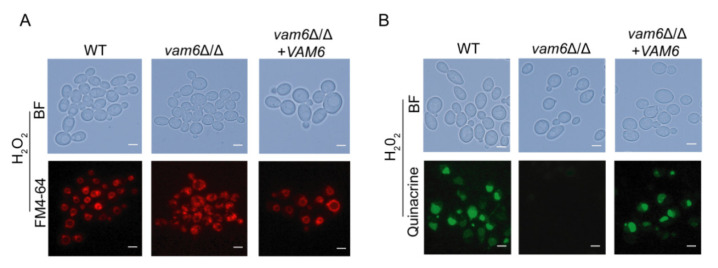
Deletion of *VAM6* has an impact on vacuolar morphology and function. (**A**) Vacuolar morphology of the strains revealed by FM4-64 staining. Vacuolar morphology was observed by a fluorescence microscopy after FM4-64 staining. Bar = 5 μm. (**B**) Vacuolar pH under oxidative stress revealed by Quinacrine staining. Bar = 5 μm.

It was reported that deletion of ScVps39 not only results in abnormal morphology and function of vacuoles, but also disrupts the integrity of the vacuole structure in *S. cerevisiae* [23]. We therefore investigated the role of Vam6 in maintaining the vacuolar structure in *C. albicans*. FM4-64 staining showed that the morphology of vacuoles in *vam6*∆/∆ strain was abnormal, and the vacuoles could not aggregate to form large vacuoles (Figure 3A). Especially, under treatment of H_2_O_2_, while the control strains maintained normal round-like vacuoles, the mutant exhibited whole-cell distribution of the vacuoles with no regular morphology (Figure 3A). These observations showed that Vam6 is involved in vacuolar fusion and maintenance of vacuolar structure. 

Acidic pH in vacuoles is required for normal vacuolar functions, e.g., degradation of proteins and damaged organelles that are transported into the vacuoles [24,25]. Vacuolar pH assays by Quinacrine staining revealed that both the WT and reconstituted strains had normal vacuolar acidic pH, leading to strong fluorescence emission in the vacuoles (Figure 3B). In contrast, *vam6*Δ/Δ did not exhibit fluorescence emission, indicating that the mutant failed to maintain acidic pH in the vacuoles. Together, these results suggested that Vam6 is required for maintaining vacuolar structures and functions in *C. albicans*.

### 3.4. Vam6 Is Required for Maintaining Mitochondrial Morphology under Oxidative Stress 

**Figure 4:**
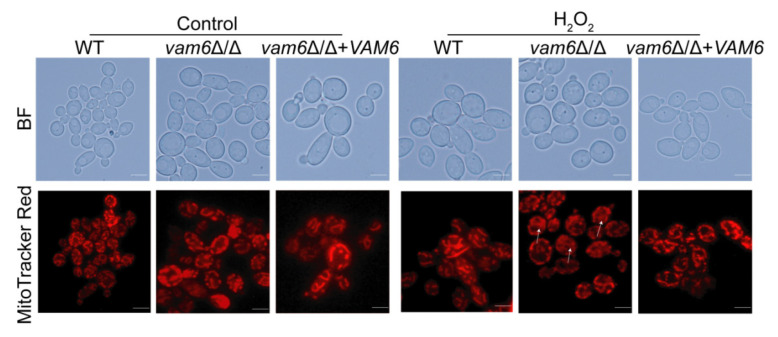
Deletion of *VAM6* impairs mitochondrial morphology under Mitochondrial morphology was abnormal under oxidative stress. Fluorescence microscopy images of the strains stained by MitoTracker Red. The cells were cultured in liquid YPD containing 5 mM H_2_O_2_ or not, stained, and observed. Bar = 5 μm.

To investigate the role of Vam6 in mitochondrial functions, the mitochondria were visualized by MitoTracker Red staining [26]. Generally, the mitochondria had a tubular structure, exhibiting a bifurcated, cross-linked network in WT under oxidative stress. Moreover, the *vam6*Δ/Δ+*VAM6* significantly restore normal mitochondrial network (Figure 4). In contrast, the mitochondria in *vam6*∆/∆ were significantly fragmented under oxidative stress (Figure 4). Therefore, these results revealed that Vam6 plays an important role in maintaining normal mitochondrial morphology under oxidative stress.

### 3.5. Deletion of VAM6 Leads to Mitochondrial Dysfunction

**Figure 5:**
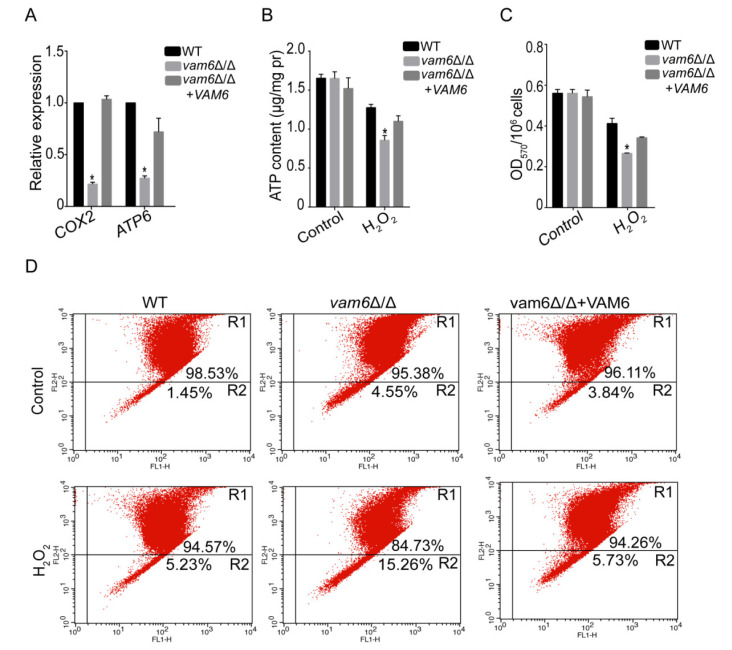
Deletion of *VAM6* affects mitochondrial functions under oxidative stress. (**A**) Effect of *VAM6* deletion on mtDNA quantity after cultured for 4 h under oxidative stress. The mtDNA encoding genes *COX2*, *ATP6* were analyzed by RT-PCR using *ACT1* as the normalization gene. (**B**) Intracellular ATP levelsmeasured by an ATP detection kit. (**C**) Impact of *VAM6* deletion on mitochondrial SDH activity. Cultures were mixed with MTT and incubated at 30 °C for 2 h, and then collected. The pellets were then resuspended in DMSO. The suspensions were centrifuged, and OD_570_ of the supernatants was measured. (**D**) Effect of *VAM6* deletion on mitochondrial membrane potential revealed by JC-1 staining and flow cytometry. The cells were collected, resuspended in PBS and incubated with JC-1 for 30 min at 37 °C. Gated region R1 represents cells with intact mitochondrial membranes and gated region R2 shows cells with loss of mitochondrial membrane potential. This value represents the mean ± SD from three replicates. * Significant difference between mutants and control strains, *p* < 0.05.

In *S. cerevisiae*, mitochondrial DNA (mtDNA, e.g., *COX2*, *ATP6*) stability affects cell growth. In this study, the role of Vam6 in maintaining mtDNA stability was explored by real-time quantitative PCR (qRT-PCR). The result showed that the copy numbers of tested mtDNA genes were significantly reduced in *vam6*Δ/Δ as compared to control strains under H_2_O_2_ conditions (Figure 5A) confirming that Vam6 was involved in maintaining mtDNA stability under oxidative stress.

Mitochondrial activity is closely related to mitochondrial ability to produce ATP [27,28]. Therefore, the effect of *VAM6* deletion on mitochondrial ATP production was detected by the ATP assay kit. As compared to the WT and reconstituted strains, the mutant had lower ATP levels under oxidative stress (Figure 5B), indicating an impairment of mitochondrial function in the mutant. Moreover, MTT assays showed that the activity of NAD(P)H-dependent enzymes, one of the markers reflecting mitochondrial function [10], was significantly reduced in *vam6*Δ/Δ as compared to control strains under H_2_O_2_ conditions (Figure 5C). 

Studies have shown that mitochondria dysfunction was closely associated with a decrease in mitochondrial membrane potential (MMP) [29]. In this study, the cationic lipid fluorescent dye JC-1 was used to study the effect of *VAM6* deletion on MMP by flow cytometry. The results showed that only 4.55% of the mutant cells showed decreased MMP, which was slightly higher than the control strains under the normal condition (Figure 5D). However, under the oxidative stress, the mutant showed higher levels of cells with decreased MMP than the control strains (15.26% versus 5.23% or 5.73%). Taken together, these data indicate that Vam6 plays a critical role in the maintenance of mitochondrial function under oxidative stress.

### 3.6. Effect of Vam6 on Oxidative Stress Response and Antioxidant Capacity

**Figure 6:**
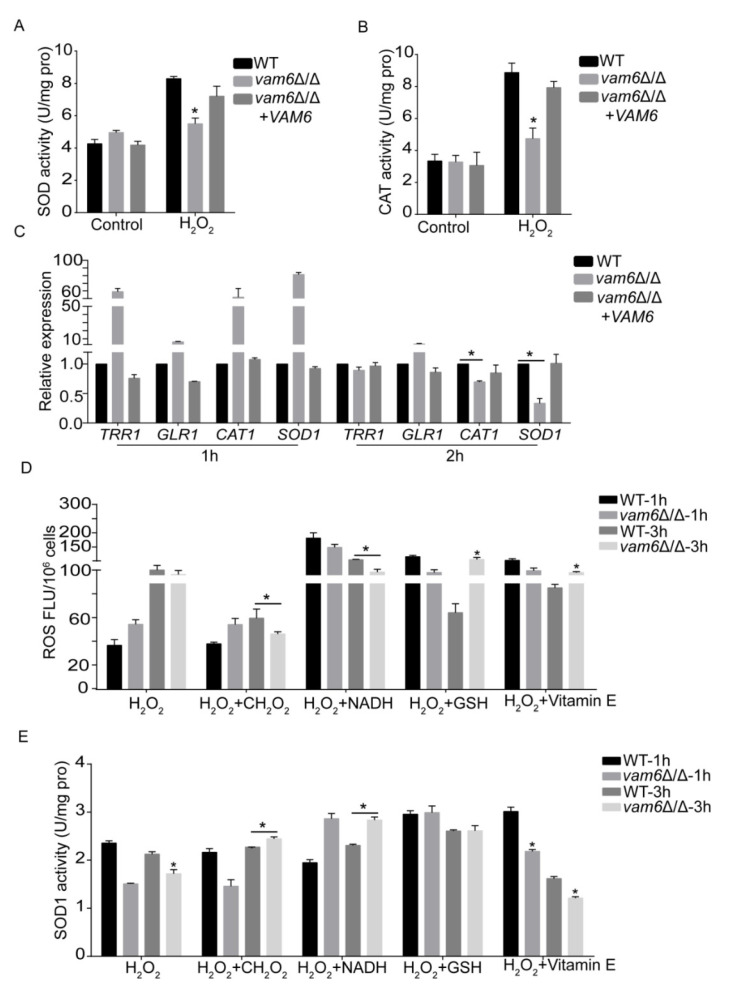
Deletion of *VAM6* affected the antioxidant capacity of *C. albicans*. (**A**,**B**) Activity of SOD (**A**) and CAT (**B**) in the strains treated by 5 mM H_2_O_2_ or not. (**C**) Expression levels of oxidative stress-responsive genes in the strains revealed by RT-PCR, with *ACT1* as the normalization gene. (**D**) Effect of antioxidants (i.e., CH_2_O_2_, NADH, reduced glutathione (GSH), and Vitamin E) on intracellular ROS levels. Cells were cultured in YPD medium containing 5 mM H_2_O_2_ plus 50 mM CH_2_O_2_, 50 μM NADH, 10 mM GSH, 10 mM Vitamin E. After 1 h, 3 h of incubation with shaking at 30 °C. ROS were determined by DCFH-DA staining. (**E**) SOD1 activity in the H_2_O_2_-treated cells. SOD1 activity of the supernatant was assessed by a SOD1 assay kit. The values represent the mean ± SD from three replicates. * indicates a significant difference between mutants and the control strains (*p* < 0.05).

Organisms use antioxidant enzymes (such as superoxide dismutases (SOD), catalase (CAT), etc.) to protect cells from oxidative damage by removing reactive oxygen species (ROS) [30]. In order to explore the mechanism of ROS accumulation in cells, the activity of SOD and CAT (encoding catalase) in the cells were measured. Enzymatic assays showed that oxidative stress caused remarkable increase in the activity of both SOD and CAT in the three strains (Figure 6A,B). As compared to the WT and reconstituted strains, the *vam6*∆/∆ mutant exhibited obvious lower activity of both enzymes under oxidative stress, with 15~20% lower SOD activity and 40~45% lower CAT activity (Figure 6A,B). Moreover, the expression level of antioxidant genes (Thioredoxin reductase (*TRR1*), Glutathione reductase (*GLR1*), Copper-zinc superoxide dismutase 1 (*SOD1*), Catalase (*CAT1*)) was detected by qRT-PCR. Under H_2_O_2_ treatment for 2 h, the expression levels of the *SOD1* gene and the *CAT1* gene were significantly reduced as compared to the control strains (Figure 6C). These results implied that *VAM6* deletion had an impact on the antioxidant system under oxidative stress.

Some reports indicated that superoxide dismutases (SODs) play an essential role in protecting cells under oxidative conditions, and it is indispensable to elimination the superoxide anion radical (O_2_^•−^) in the cells [31]. The SOD1 (Cu^2+^/Zn^2+^), SOD2 (Mn-SOD), SOD3 (Fe-SOD) were intracellular enzymes, while SOD4, SOD5, and SOD6 were GPI-anchored superoxide dismutase that distribute on the cell wall surface [32]. Studies have shown that SOD1 and SOD2 can remove superoxide radical (O_2_^•−^) in eukaryotic cells [32]. In order to further study the mechanism of the SOD activity decrease in the mutant, the SOD1 (Copper/zinc superoxide dismutase) enzyme activity and ROS accumulation levels were examined. It can be seen that the addition of antioxidants, i.e., formic acid (CH_2_O_2_) and NADH, could effectively recover SOD1 enzyme activity and reduce ROS accumulation level in *vam6*∆/∆ under oxidative stress (Figure 6D,E). However, there was no significant change of SOD2 activity (results not shown). Our findings suggest that deletion of *VAM6* reduces the antioxidant capacity of *vam6*∆/∆ by reducing the SOD1 enzyme activity.

### 3.7. Deletion of VAM6 Impairs Morphogenesis in C. albicans

**Figure 7:**
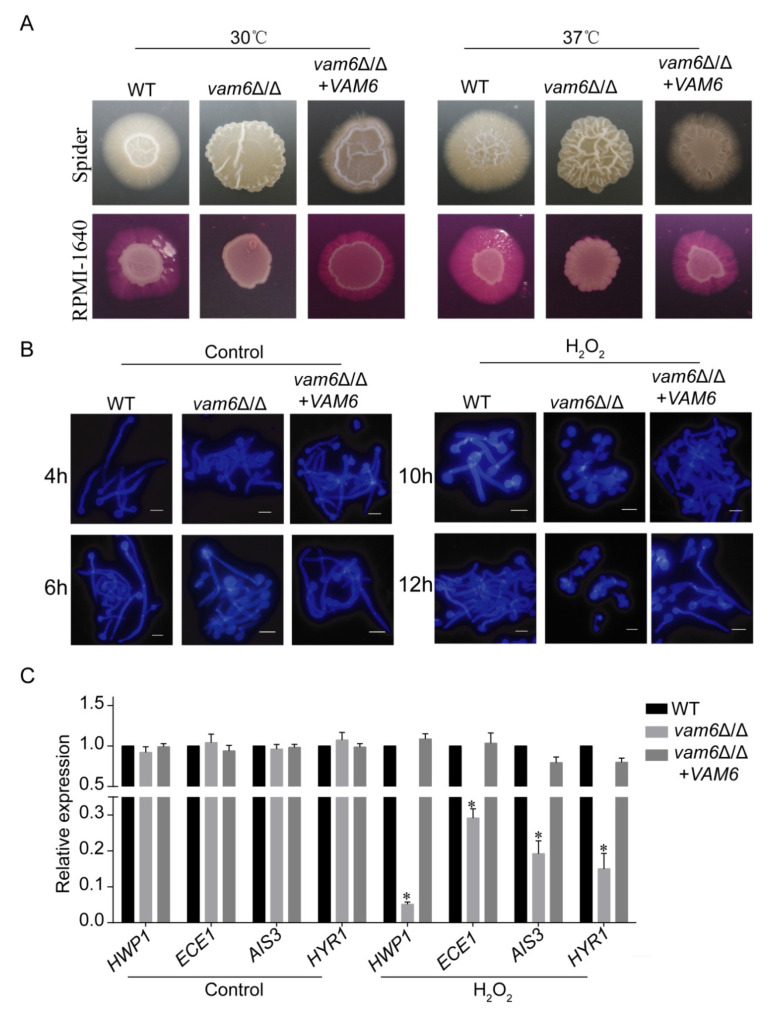
The effect of *VAM6* deletion on hyphal development of *C. albicans*. (**A**) Hyphal development on the solid hypha-inducing media. The strains were spotted on the indicated agar plates and incubated at 30 °C or 37 °C for five days. (**B**) Hyphal development in the liquid RPMI-1640 medium in the absence of (Control) or in the presence of H_2_O_2_. Left, the cells were incubated in the medium at 37 °C for 2 h, 4 h, or 6 h, and stained by CFW for observation. Right, the strains were incubated in liquid RPMI-1640 containing 5 mM H_2_O_2_ at 37 °C for 10 h or 12 h, and stained by CFW then observed. Bar = 5 μm. (**C**) Effect of *VAM6* deletion on expression of hypha related genes. Cells were collected after treated by 5 mM H_2_O_2_ in liquid RPMI-1640 medium for 1 h, and then RNA was extracted. The expression of *AIS3*, *ECE1*, *HYR1*, and *HWP1* were measured by RT-PCR using *ACT1* as the normalization gene. The relative measurement was respectively under control and H_2_O_2_ conditions. This value represents the mean ± SD with three replicates. * indicates a significant difference between the mutant and the control strains (*p* < 0.05).

Studies have found that morphogenesis (e.g., hyphal development) is very important for the virulence of *C. albicans* [33]. There were many factors that affect the morphogenic ability of *C. albicans*. However, the effect of vCLAMPs on morphogenesis has not been reported in *C. albicans.* After culturing at 30 °C and 37 °C, unlike the control strains forming wrinkled colonies with obvious hyphal extension, the *vam6*Δ/Δ mutant formed smooth colonies without wrinkles and hyphae around the colony (Figure 7A). Moreover, the WT, *vam6*Δ/Δ, and reconstituted strains could all form hyphal cells in RPMI-1640 medium (Figure 7B). However, under the oxidative stress condition, while the WT and reconstituted strains could form hyphae after 10~12 h, the mutant failed to do so. These results confirmed that Vam6 plays an important role in hyphal development in *C. albicans* under oxidative stress conditions. 

qRT-PCR experiments were performed to detect the expression of genes related to hyphae development in all strains. Under oxidative stress, the expression levels of hypha-relating genes were reduced in *vam6*Δ/Δ strain (Figure 7C and Figure S4). These genes included the hyphal cell wall gene *HWP1*, the biofilm formation-related gene *ECE1*, the cell membrane-related gene *HYR1*, and the adhesion-related gene *ALS3* [34,35]. Above all, these results indicated that Vam6 is involved in hyphal development under oxidative stress, which is associated with regulation of expression of hypha-related genes.

### 3.8. Deletion of VAM6 Dramatically Reduces the Virulence of C. albicans

The above results revealed that Vam6 is involved in maintaining both vacuolar and mitochondrial functions, together with regulation of morphogenesis, all of which are associated with the virulence of *C. albicans.* Therefore, we speculated that *VAM6* deletion may reduce the pathogenicity of *C. albicans*. We used both the *C. albicans*-macrophage interaction model and the mouse systemic infection model to investigate the function of Vam6 in the pathogenesis of *C. albicans*. Since *URA3* can influence the virulence of *C. albicans*, we reintroduced *URA3* to the strains by the plasmid pLUBP to ensure the accuracy of virulence experiment results [36]. These mutants were named WT^a^, *vam6*∆/∆^a^, *vam6*∆/∆+*VAM6*^a^.

**Figure 8:**
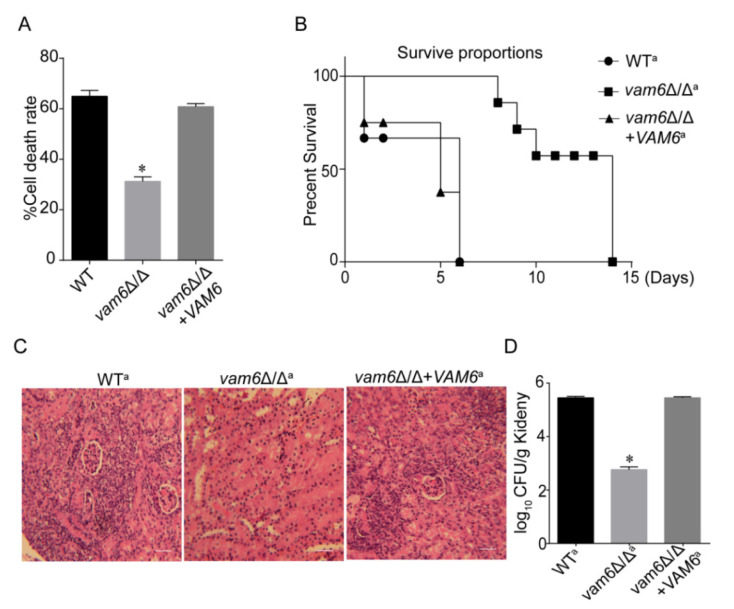
Effect of *VAM6* deletion on virulence of *C. albicans*. (**A**) Macrophage-killing capacity of the strains WT^a^, *vam6*Δ/Δ^a^, *vam6*Δ/Δ+*VAM6*^a^. (**B**) Survival rate of the mice infected by the strains. (**C**) Histopathological images of the kidneys infected by the strains. Bar = 50 μm. (**D**) Fungal burden in the kidney suffering from infection by the strains after five days. * indicates significant difference between the control strains and the mutant (*p* < 0.05). Values represent mean ± SD.

The macrophage-killing ability of *C. albicans* was determined by the *C. albicans*-macrophage interaction model. We found that the killing capacity of *vam6*∆/∆ was reduced, compared the control strains (Figure 8A). While the control strains could kill 60% macrophages, the mutant only killed ~30% macrophages (Figure 8A). Therefore, deletion of *VAM6* severely attenuated the capacity of the fungal cells to kill macrophages.

To further test the effect of *VAM6* deletion on the virulence of *C. albicans*, a systematic mouse infection model was further used. While all mice infected by the WT and reconstituted strains died in five days, all the mice infected with *vam6*∆/∆^a^ survived in these days, and died after 15 days, revealing that deletion of *VAM6* prolonged the survival time of the fungus-infected mice. Moreover, it was found that the mouse kidneys infected by the mutant had lower inflammation degree (Figure 8C and Figure S5), and exhibited much lower fungal burden than the kidneys infected by the control strains (Figure 8D). Together, these results confirm that Vam6 plays an important role in the pathogenicity of *C. albicans* cells. 

## 4. Discussion

Previous studies revealed that ScVps39 is a vCLAMP protein in *S. cerevisiae* [17]. However, the cellular biological functions of *C. albicans* Vam6, the homolog of ScVps39, have been less widely studied. In this study, we confirmed that Vam6 is a core vCLAMP protein in *C. albicans.* Based on fluorescence microscopy techniques and immunotransmission electron microscope (IEM), we found that the Vam6 protein localized on the connection site of mitochondria and vacuoles in *C. albicans* (Figure 1). Furthermore, deletion of *VAM6* significantly reduced the number of vCLAMPs. Above all, we successfully confirmed the role of Vam6 in vCLAMP formation of *C. albicans*. Our work provides a new insight in vCLAMPs of this pathogen.

In this study, deletion of *VAM6* caused a defect in vacuolar fusion in *C. albicans* (Figure 3A). In recent years, some research has found that deletion of ScVps39 causes vacuoles to form numerous small vesicles in *S. cerevisiae*, which is consistent with the findings of our study [15,17]. It was found that loss of CmVps39 also made the vacuoles smaller in *Coniothyrium minitans*. Vacuoles can be formed by aggregation of small vesicles to form large vacuoles, or by split of large vacuoles in *C. albicans* [15,17,37]. Fusion and division of vacuoles were indispensable to maintain normal cell viability [38]. ScVps39, as a vacuolar Rab/Ypt effector, cooperates with Ypt7p to mediate and promote vacuolar fusion in *S. cerevisiae* [39]. Those results indicated that ScVps39 is an important factor for vacuolar fusion. It has been reported that vacuole have the function of adjusting pH balance in *S. cerevisiae* [40]. Herein, we found that the *vam6*∆/∆ mutant failed to maintain acidic pH under oxidative stress. Some reports point that acidic environment of vacuoles was very important to maintain the enzyme activity in *C. albicans* [25]. We speculate that the abnormal vacuolar pH is due to the morphology change of the vacuoles, which results in the decrease of the acid-base balance enzyme activity under oxidative stress. The study revealed that the vCLAMP protein Vam6 was necessary to maintain vacuolar acidity under oxidative stress. In addition, our data leave open the question of the *VAM6* deletion significantly increasing the sensitivity to copper ions in *C. albicans*, which was different to the findings in *S. cerevisiae* [17]. The mechanism needs further study. In summary, these results suggested that Vam6 plays a vital role in maintaining vacuole morphology and functions of *C. albicans*.

The mitochondrion is the antioxidant center of eukaryotic cells. It was not only the main ROS-generating site, but also the ROS removing site [41]. In recent years, some researches indicated that the mitochondria were attacked by ROS when the cell’s antioxidant capacity was compromised. ROS caused oxidative damage to the mitochondria by reduction of cardiolipin synthesis, degradation of mtDNA and inactivation of mitochondrial proteins [41]. The results of this study indicate that deletion of *VAM6* caused abnormal mitochondrial morphology (Figure 4). Moreover, the expression levels of mtDNA [13,42], e.g., *COX2*, *ATP6* were significantly reduced under oxidative stress (Figure 5A). Mitochondrial membrane potential and SDH activity were also dramatically reduced in *vam6*Δ/Δ mutant [43,44]. Those results indicated that the vCLAMP core protein Vam6 is important to maintenance of mitochondrial functions under oxidative stress. 

Some studies have shown that accumulation of dysfunctional mitochondria may cause accumulation of ROS in cells [41,45]. Interestingly, deletion of the *VAM6* gene caused a significant increase of ROS in *vam6*Δ/Δ. We speculated that high levels of ROS may be due to the reduced ROS-scavenging enzyme activity. In this study, the expression of ROS scavenging enzymes (SOD and CAT) and related genes were significantly decreased under oxidative stress in *vam6*Δ/Δ [46]. Some reports pointed out that once *C. albicans* successfully infects host tissues, the fungal oxidative stress response system is activated, and the secretion of anti-oxidant enzymes becomes important to resist oxidative stress. Among them, superoxide dismutases (SODs) play a critical role in ROS removal. 

Studies have shown that SOD1 and SOD2 exist in eukaryotic cells [32], and remove superoxide anions of intracellular. Therefore, we speculate that the significant accumulation of ROS was due to decreased SOD enzyme activity in the mutant. It was found that only the addition of superoxide scavengers, such as CH_2_O_2_ and NADH, can reduce the accumulation of ROS under oxidative stress [47]. However, there was no significant change of SOD2 activity. Therefore, the present research work brought about a discovery that the abnormal antioxidant capacity was due to a decrease of SOD1 enzyme activity in *vam6*Δ/Δ. These findings confirmed that the vCLAMP core protein Vam6 was of significance to oxidative stress resistance.

Herein, Vam6 was also involved in hyphal development. Under oxidative stress, deletion of *VAM6* led to significant abnormalities in hyphal development of *C. albicans*. The above findings can be summarized as follows. (1) As a key organelle of the fungus, the vacuoles participate in long-distance transportation of nutrients within the hypha, and can also regulate the expansion and branch formation of the fungal hypha [48,49]. This research found that deletion of *VAM6* caused abnormal vacuolar morphology, and led to a defect in vacuolar fusion. Therefore, we speculate that the process of intracellular nutrient transport involved in cells was blocked, which makes it difficult to transport nutrients to the growth point for hyphal development. (2) The mitochondria are the core of energy production, and the main place for aerobic respiration in cells. Some reports found that mycelium development requires mitochondria to provide energy. For example, studies have shown that mitochondrial ATP synthases were involved in regulating ascospore germination and sexual reproduction in *Podospora anserina* [50]. The results reported here support that mitochondrial morphology was abnormal and the ridge was loose in *vam6*∆/∆ under oxidative stress. The results presented here suggested that the mitochondrial functions were impaired, leading to decreased energy metabolism required for hyphal development. (3) Cell viability affects hyphal development of *C. alibcans*. In this study loss of Vam6 rendered the cell viability under oxidative stress. Therefore, we speculate that the action of H_2_O_2_ did not lead to hyphal development in *vam6*∆/∆ mutant. The synergistic effect of various virulence factors of *C. albicans* leads to an increase or decrease of its pathogenicity. When an imbalance of certain factors occurs, the ability of the pathogen to infect the host will be weakened. As a core protein of vCLAMPs, Vam6 plays an important role in maintaining oxidative stress resistance, hyphal development, and virulence of *C. albicans*. 

## 5. Conclusions

This study revealed that the vCLAMP protein Vam6 is critical for the formation of the vCLAMP structure, oxidative stress tolerance, and maintenance of mitochondrial and vacuolar functions under oxidative stress. The wide-ranging functions of Vam6 make it an important factor required for the maintenance of the virulence of this pathogen. Therefore, vCLAMPs may be a novel target of antifungal therapies for the treatment of *C. albicans*-related infections.

## Data Availability

Data is contained within the article or Supplementary Material.

## Supplementary Materials

The following are available online at https://www.mdpi.com/2076-3921/10/1/136/s1, Figure S1: Construction and identification of *vam6*Δ/Δ mutants, Figure S2: Localization of Csp37 was observer in WT-*CSP37-GFP*, Figure S3: Ions sensitivity analysis of the *vam6*Δ/Δ mutant, Figure S4: Effect of *VAM6* deletion on expression of hypha related genes, Figure S5: Quantify the tissue damage of kidneys, Table S1: Strains and plasmids used in this study, Table S2: Primers used in this study. References [1,51,52] are cited in the Supplementary Material.
