# Correction: Mao et al. Vacuole and Mitochondria Patch (vCLAMP) Protein Vam6 Is Involved in Maintenance of Mitochondrial and Vacuolar Functions Under Oxidative Stress in *Candida albicans*. *Antioxidants* 2021, *10*, 136

**DOI:** 10.3390/antiox15030375

**Published:** 2026-03-17

**Authors:** Xiaolong Mao, Li Yang, Yingzheng Liu, Congcong Ma, Tianyu Ma, Qilin Yu, Mingchun Li

**Affiliations:** Key Laboratory of Molecular Microbiology and Technology, Ministry of Education, College of Life Science, Nankai University, Tianjin 300071, China; fudan671@163.com (X.M.); 18783958821@163.com (L.Y.); liuwenbo129@163.com (Y.L.); mcmacongcong@163.com (C.M.); matianyu3317658@163.com (T.M.); yuqilin7007@163.com (Q.Y.)

There was a mistake in the published paper [1]. In Figure S3 in the Supplementary Materials, 10 mmol/L Mn^2+^ was inadvertently duplicated from 50 mmol/L Mg^2+^ in Figure S3 during the image-organizing process. The correct version of Figure S3 is presented below:

The authors state that the scientific conclusions are unaffected. This correction was approved by the Academic Editor. The original publication has also been updated.

## Figures and Tables

**Figure S3:**
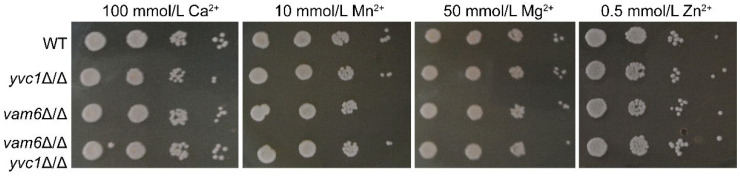
Ions sensitivity analysis of the *vam6*Δ/Δ mutant. Cells were cultured overnight in liquid YPD and spotted on YPD plates containing 100 mM Ca^2+^, 10 mM Mn^2+^, 50 mM Mg^2+^, or 0.5 mM Zn^2+^. The plates were then cultured and photographed.
