# Supplementary material for: Vacuole and Mitochondria Patch (vCLAMP) Protein Vam6 Is Involved in Maintenance of Mitochondrial and Vacuolar Functions under Oxidative Stress in Candida albicans
Source: Antioxidants (Basel). 2021 Jan 19;10(1):136. doi: 10.3390/antiox10010136 (PMC7835768; doi:10.3390/antiox10010136)
Supplement: Supplementary file 1 [file antioxidants-10-00136-s001.pdf]

Supplementary Materials:

Supplementary Figures

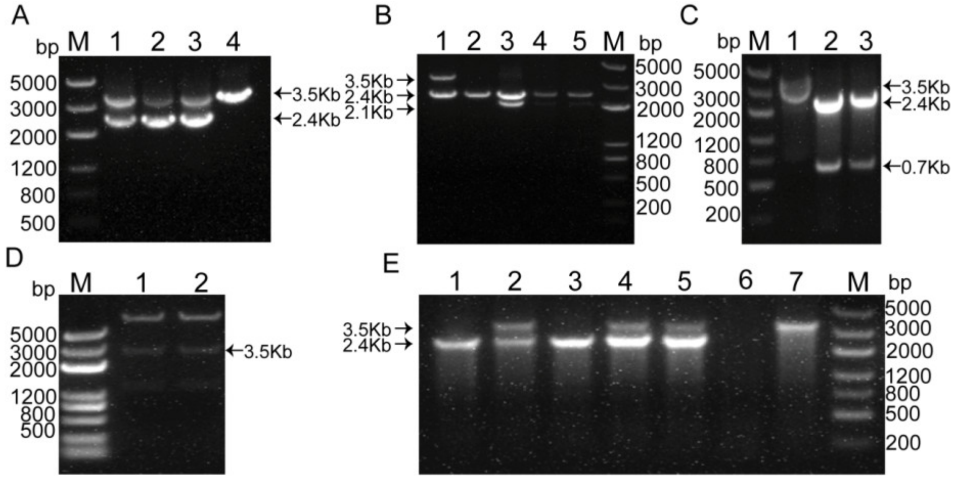

Figure S1. Construction and identification of *vam6*Δ/Δ mutants. (**A**) Identification of *vam6*Δ/*VAM6*. Line 1-4, the amplified fragment. (**B**) Identification of *vam6*Δ/Δ (URA3). Line 1-5, the amplified fragment. (**C**) Identification of *vam6*Δ/Δ (-*URA3*). Line 1-3, the amplified fragment. (**D**) Enzyme digestion identification of pDDB78-*VAM6*. (**E**) PCR identification of *VAM6* complemented stains.

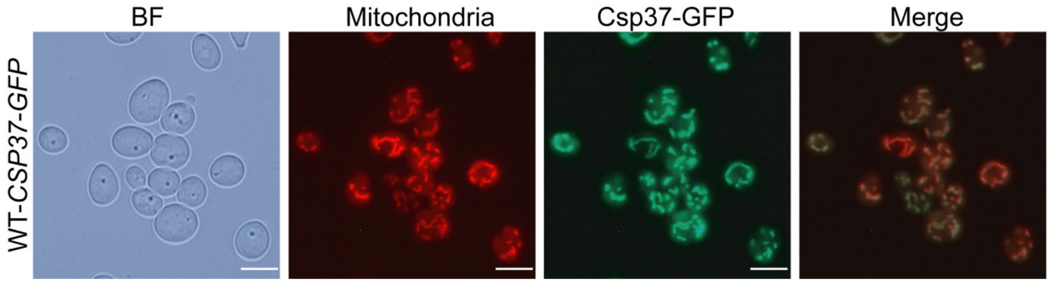

Figure S2. Localization of Csp37 was observer in WT-*CSP37-GFP*. Csp37-GFP: Csp37 localization, green fluorescence. Mito Tracker Red: Mitochondrial localization, red fluorescence. BF: Bright field.

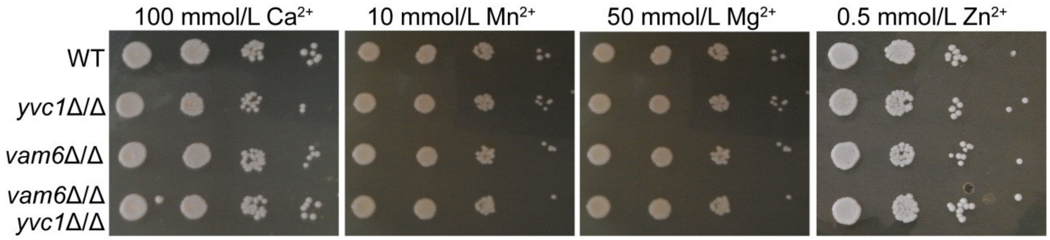

Figure S3. Ions sensitivity analysis of the *vam6*Δ/Δ mutant. (A) Cells were overnight cultured in liquid YPD, spotted on YPD plates containing 100 mM Ca^{2+}, 10 mM Mn^{2+}, 50 mM Mg^{2+} , or 0.5 mM Zn^{2+}. The plates were then cultured and photographed.

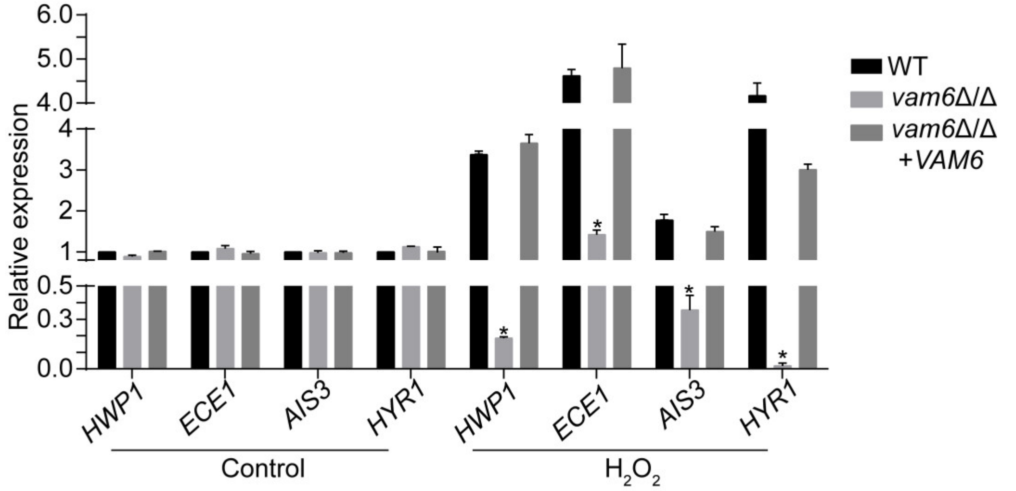

Figure S4. Effect of *VAM6* deletion on expression of hypha related genes. Cells were collected after treated by 5 mM H_2O_2 in liquid RPMI-1640 medium for 1 h, and then RNA was extracted. The expression of *AIS3*, *ECE1*, *HYR1*, and *HWP1* were measured by RT-PCR using *ACT1* as the normalization gene. This value represents the mean ± SD with three replicates. * indicates a significant difference between the mutant and the control strains ($p < 0.05$).

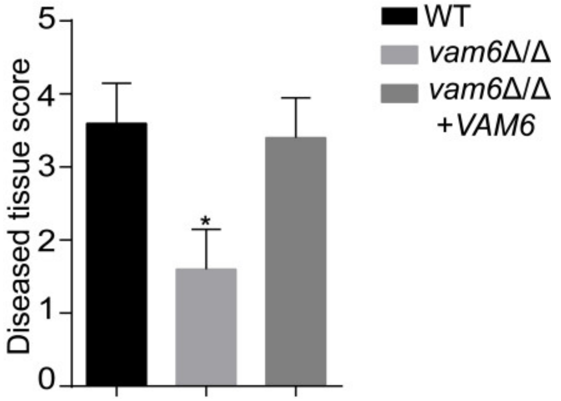

Figure S5. Quantify the tissue damage of kidneys. Take 5 fields for each histopathological images, calculate the ratio of the area of the lesion to the entire field in each field, 0 points for no disease, 1 point for <25%, 2 points for 25%-50%, 3 points for 50%-75 %, 4 points for >75%.* indicates a significant difference between the mutant and the control strains ($p < 0.05$).

Supplementary Tables:

Table 1. Strains and plasmids used in this study.

Strain/ Plasmid	Description	Source/Purpose
BWP17	*ura3Δ::limm434/ura3Δ::limm434 his1::hisG/his1::hisG arg4::hisG/arg4::hisG*	[51]
BWP17[a]	*URA3/ura3Δ::limm434his1::hisG/his1::hisG arg4::hisG/arg4::hisG*	This study
*vam6*Δ/Δ (+*URA3*)	*ura3Δ::limm434/ura3Δ::limm434his1::hisG/his1::hisGarg4:: hisG/arg4::hisG vam6::ARG4/vam6::dpl200-URA3*	This study
*yvc1*Δ/Δ	*ura3Δ::limm434/ura3Δ::limm434 his1::hisG/his1::hisG arg4::hisG/arg4::hisG yvc1::ARG4/yvc1::dpl200*	[1]
*vam6*Δ/Δ	*ura3Δ::limm434/ura3Δ::limm434 his1::hisG/his1::hisG arg4::hisG/arg4::hisG vam6::ARG4/vam6::dpl200*	This study
*vam6*Δ/Δ[a]	*URA3/ura3Δ::limm434his1::hisG/his1::hisGarg4::hisG/arg4: :hisG vam6::ARG4/vam6::dpl200*	This study
*vam6*Δ/Δ*yvc1*Δ/Δ	*ura3Δ::limm434/ura3Δ::limm434 his1::hisG/his1::hisG*	This study

	arg4::hisG/arg4::hisG yvc1::ARG4/yvc1::dpl200, vam6::dpl200/vam6::dpl200	
*vam6*Δ/Δ+*VAM6*	*ura3Δ::limm434/ura3Δ::limm434 his1::hisG/his1::hisG arg4::hisG/arg4::hisG vam6::ARG4/vam6::dpl200,* pDDB78-VAM6	This study
*vam6*Δ/Δ+*VAM6*[a]	*URA3/ura3Δ::limm434his1::hisG/his1::hisGarg4::hisG/arg4::hisG vam6::ARG4/vam6::dpl200,* pDDB78-*VAM6*	This study
*vam6*Δ/Δ-*CSP37-GFP*	*ura3Δ::limm434/ura3Δ::limm434 his1::hisG/his1::hisG arg4::hisG/arg4::hisG vam6::ARG4/vam6::dpl200 CSP37/CSP37-GFP::URA3*	This study
WT-*VAM6-GFP*	*ura3Δ::limm434/ura3Δ::limm434 his1::hisG/his1::hisG arg4::hisG/arg4::hisG VAM6/VAM6-GFP::URA3*	This study
WT-*VAM6*	*ura3Δ::limm434/ura3Δ::limm434 his1::hisG/his1::hisG arg4::hisG/arg4::hisG VAM6/VAM6::URA3*	This study
WT-*CSP37-GFP*	*ura3Δ::limm434/ura3Δ::limm434 his1::hisG/his1::hisG arg4::hisG/arg4::hisG CSP37/CSP37-GFP::URA3*	This study
Plasmid		
pRS-ArgΔ*Spe*I	Amp^r *ARG4*	[51]
pDDB57	Amp^r *URA3*	[51]
pDDB78	Amp^r *TRP1 HIS1*	[51]
pLUBP	Amp^r *IRO1-URA3*	[52]
pDDB78-*VAM6*	Amp^r *VAM6 TRP1 HIS1*	This study
pAu34M-GFP	Amp^r P_{ACT1}*-GFP URA3*	This study
pAu34M–*VAM6-GFP*	Amp^r P_{ACT1}*-VAM6-GFP URA3*	This study

[a] Strain that *URA3* was reintroduced at its common locus.

Table 2. Primers used in this study.

Primer	Sequence (5′→3′)	Function
VAM6-5DR	AAATTAAGGGGAATT-AGGGTTAACAAAATTACACGTCC-TACAGATGCATTGTTTTGTTTATTTCCCAGTCAC-GACGTT	Gene knockout
VAM6-3DR	AATATGAGACTTTTTAAAAATTACTTGTATGA-TAAGATATATATTTAATTAAACTAACGTTGGAATT-GTGAGCGGATA	Gene knockout
VAM6-5det	AAATGGAAATCGTAGAAAGT	Genetic validation
VAM6-3det	CTTAGATGTGTTGTTGTTTT	Genetic validation
VAM6-5com	ATATAATCATTCAAAATGGTAATGAATATAGATGT	Complement gene
VAM6-3com	ATATAATCATTCAAAATGGTAATGAATATAGATGT	Complement gene
VAM6-5GFP	GGACTAGTATGGTTTCAAAAGGTGAAG	Transcriptional fusion
VAM6-3GFP	TTCCAGAATTTCACTCTTATTTATA-TAATTCATCCATA	Transcriptional fusion
COX2-5RT	TAGATGTACCTACACCTTGA	RT-PCR
COX2-3RT	CCTATAGAGTCAACAAAATC	RT-PCR
NAD2-5RT	ACATACATAGTTTATATCGC	RT-PCR
NAD2-3RT	GAGAGTAAGATTGTAATTCA	RT-PCR
NAD5-5RT	TACTTGAATTAGTCTAGGTG	RT-PCR
NAD5-3RT	CTACAAACTCTCAACCAATG	RT-PCR

ATP6-5RT	TTCACCTTTAGATCAATTCG	RT-PCR
ATP6-3RT	AAACTAAATGATACAATAGC	RT-PCR
SCR1-5RT	CTGCTCGTGTACCTGCTGTT	RT-PCR
SCR1-3RT	CTTCCCTCCAGTGGTTATGCT	RT-PCR
URA3-5inner	CGCGGGATTTGGATGGTAT	URA test
URA3-3inner	TCTTGGCTCTTGGTTGGTG	URA test
GLR1-5RT	GGAGATTTCGATTGGGCTAA	RT-PCR
GLR1-3RT	GACTTCAACTTCACCTTCAG	RT-PCR
TRR1-5RT	TGGAGGATCTGAATTGATGG	RT-PCR
TRR1-3RT	CACCAGTAGCAATGATAACG	RT-PCR
CAT1-5RT	TCCAGAACCATTTGCCACTCA	RT-PCR
CAT1-3RT	GGAACCTTTAGCGTGGACAAC	RT-PCR
SOD1-5RT	GCTGTTGTCAGAGGTGATTCA	RT-PCR
SOD1-3RT	TGAAATGAGGACCAGCAGA	RT-PCR
HWP1-5RT	TGTCTACACTACATTCTGTC	RT-PCR
HWP1-3RT	AGGAATAGATGGTTGTGAAC	RT-PCR
ECE1-5RT	CCAAGCACCTACTGTTCC	RT-PCR
ECE1-3RT	GATACCAGCAACAACAGAAT	RT-PCR
ALS3-5RT	CTCATTACACCAACCATACA	RT-PCR
ALS3-3RT	GGATTCTGTGGTTGTAGTAT	RT-PCR
HYR1-5RT	CTCATTACACCAACCATACA	RT-PCR
HYR1-3RT	TCCAGAACCAGAGCCATC	RT-PCR
ACT1-5RT	GGTAGACCAAGACATCAAGG	RT-PCR
ACT1-3RT	CCGTGTTCAATTGGGTATCT	RT-PCR
